# An expanded phenotype centric benchmark of variant prioritisation tools

**DOI:** 10.1002/humu.24362

**Published:** 2022-03-09

**Authors:** Denise Anderson, Timo Lassmann

**Affiliations:** ^1^ Telethon Kids Institute Precision Health Computational Biology The University of Western Australia Subiaco Western Australia Australia

**Keywords:** dbNSFP, disease, human phenotype ontology, phenotype, variant prioritization

## Abstract

Identifying the causal variant for diagnosis of genetic diseases is challenging when using next‐generation sequencing approaches and variant prioritization tools can assist in this task. These tools provide in silico predictions of variant pathogenicity, however they are agnostic to the disease under study. We previously performed a disease‐specific benchmark of 24 such tools to assess how they perform in different disease contexts. We found that the tools themselves show large differences in performance, but more importantly that the best tools for variant prioritization are dependent on the disease phenotypes being considered. Here we expand the assessment to 37 tools and refine our assessment by separating performance for nonsynonymous single nucleotide variants (nsSNVs) and missense variants (i.e., excluding nonsense variants). We found differences in performance for missense variants compared to nsSNVs and recommend three tools that stand out in terms of their performance (BayesDel, CADD, and ClinPred).

1

Next‐generation sequencing for clinical diagnosis of genetic diseases is routinely used, however, filtering and interpreting the tens of thousands (whole exome sequencing) or millions (whole genome sequencing) of variants identified by these approaches remains challenging (Caspar et al., 2018). Variant prioritization tools assist in this task by predicting the likely pathogenicity of variants in silico, thereby enabling ranking and filtering of variants. We previously performed a benchmark study of 24 variant prioritization tools and reported that performance differs depending on the disease phenotype and recommended use of five top performing tools (Anderson & Lassmann, 2018). Here we present an update to our benchmark that incorporates additional variant prioritization tools added to the latest version of dbNSFP (Liu et al., 2020), increasing the number of assessed tools to 37. Furthermore, we refined our assessment by considering the performance of tools for nonsynonymous single nucleotide variants (nsSNVs) and missense variants (i.e., excluding nonsense variants) separately. In total, for missense variants we tested 37 tools across 4890 disease phenotypes and for nsSNVs we tested 22 tools across 5723 disease phenotypes.

Performance of the variant prioritization tools was assessed through creation of disease specific benchmark datasets. To create these datasets we (1) used terms for human phenotypic abnormalities from the Human Phenotype Ontology (HPO) resource (Köhler et al., 2014), (2) obtained the genes associated with each HPO term from the disease to gene mapping tool Phenolyzer (Yang, Robinson, & Wang, 2015) and (3) obtained the pathogenic variants residing in these genes from ClinVar (Landrum et al., 2016). For each HPO term, performance of tools was based on how well they could discriminate pathogenic variants from a set of benign variants (Niroula & Vihinen, 2019) based on the area under the precision‐recall curve (auPRC) which is suitable for inherently unbalanced data (i.e., the ratio of pathogenic to benign variants is small). We also assessed each tool based on the proportion of ClinVar pathogenic variants contained in the top 25 variants after ranking by predicted pathogenicity (PP25).

We categorized the variant prioritization tools into those that predict pathogenicity based primarily on (1) conservation scores derived from sequence alignments, (2) machine learning classifiers incorporating a diverse set of functional genomic features and (3) ensemble methods that incorporate pathogenicity scores from a number of variant prioritization tools. We used 16 conservation scores (bStatistic, FATHMM, GERP++, LIST‐S2, LRT, MutationAsssessor, phastCons17way‐primate, phastCons30way‐mammalian, phastCons100way‐vertebrate, phyloP17way‐primate, phyloP30way‐mammalian, phyloP100way‐vertebrate, PROVEAN, SIFT, SIFT4G and SiPhy), 15 machine learning scores (CADD, DANN, DEOGEN2, Eigen, Eigen‐PC, fathmm‐MKL, fathmm‐XF, fitCons‐i6, GenoCanyon, MPC, MutationTaster, PolyPhen2‐HDIV, PolyPhen2‐HVAR, PrimateAI, and VEST4) and 6 ensemble scores (BayesDel with allele frequency, BayesDel without allele frequency, ClinPred, MetaLR, MetaSVM, and REVEL). Though our focus is on classification of nsSNVs, a small number of these tools (BayesDel, CADD, MutationTaster2, PROVEAN, and SIFT) also classify insertion/deletion variants (InDels) which may be relevant for the disease under study.

Not all variant prioritization tools predict pathogenicity of nonsense variants, hence we evaluated performance for nsSNVs and missense variants separately. For missense variants, the top performing tools based on the auPRC included all of the ensemble scores (BayesDel_addAF, BayesDel_noAF, ClinPred, MetaLR, MetaSVM, and REVEL) and two machine learning scores (DEOGEN2 and VEST4) (Figure 1a and Table S1). The types of tools that perform well is more mixed when considering the PP25, with three conservation scores (LRT, phastCons100way, and SIFT), two machine learning scores (MutationTaster and Polyphen2‐HDIV) and one ensemble score (BayesDel_addAF) being the best performers (Figure S1a and Table S2). For nsSNVs, the top performing tools based on the auPRC included both ensemble scores (BayesDel_addAF and BayesDel_noAF) and four of the machine learning scores (CADD, Eigen, Eigen‐PC, and VEST4) (Figure 1b and Table S3). Of note, CADD, Eigen and Eigen‐PC were overall weak performers when prioritizing missense variants but were excellent at prioritizing nsSNVs. Again, for PP25, performance is mixed with three conservation scores (LRT, phastCons30way, and phastCons100way), two machine learning scores (CADD and MutationTaster) and two ensemble scores (BayesDel_addAF and BayesDel_noAF) showing very strong performance (Figure S1b and Table S4). The 50 HPO terms with the most variable performance across the tools for the auPRC and PP25 are shown for both missense variants and nsSNVs in  Figures S2 through S5.

**Figure 1:**
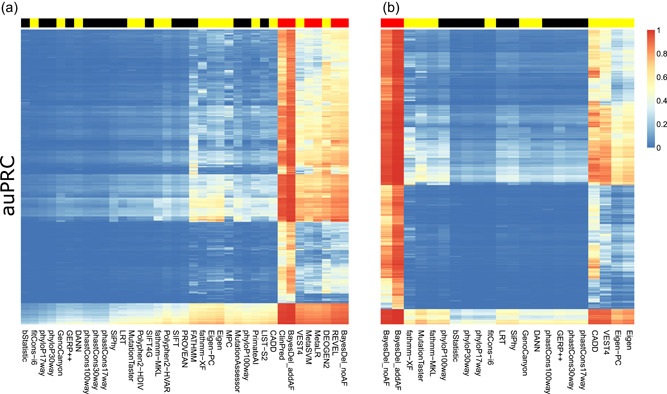
Heatmaps showing performance (auPRC) of variant prioritization tools for missense variants (a) and nsSNVs (b). Color coding of columns is based on the method used to predict pathogenicity, where black = conservation scores, yellow = machine learning scores and red = ensemble scores. Hierarchical cluster analysis with Euclidean distance and complete agglomeration was used to cluster both the tools and the HPO terms. HPO, Human Phenotype Ontology

Next, we examined performance of the top performing tools across different disease contexts. We limited this to the auPRC as there was more variability in performance across the HPO terms in comparison to the PP25 where performance was strong for most terms. We included four top level HPO terms and their descendant terms. The four top level terms were Abnormality of metabolism/homeostasis (HP:0001939), Abnormality of the immune system (HP:0002715), Abnormality of the nervous system (HP:0000707) and Neoplasm (HP:0002664).

BayesDel_addAF was clearly the strongest performer across the four top level HPO terms for missense variants, with median auPRC values ranging from 0.8 to 0.94 (Figure 2). ClinPred was the second best performer for missense variants (median auPRC range: 0.65–0.82), however, the interquartile range (IQR) was wider than that seen for BayesDel_addAF. Again, for nsSNVs, BayesDel_addAF was the best performer and auPRCs were higher and IQRs smaller than those seen for missense variants (median auPPRC range: 0.93–0.99). For both BayesDel_addAF and BayesDel_noAF, performance was stronger for nsSNVs compared to missense variants across all four top level terms. This is in contrast to VEST4, the only other tool with scores for both missense variants and nsSNVs, where performance was similar for Abnormality of metabolism/homeostasis and Abnormality of the immune system but improved for Abnormality of the nervous system and Neoplasm. Though CADD was not a top performer for missense variants, it did exhibit strong performance for nsSNVs for terms associated with Neoplasm (median auPRC = 0.83) and moderate performance for the other three top level HPO terms (median auPRC range: 0.48–0.61).

**Figure 2:**
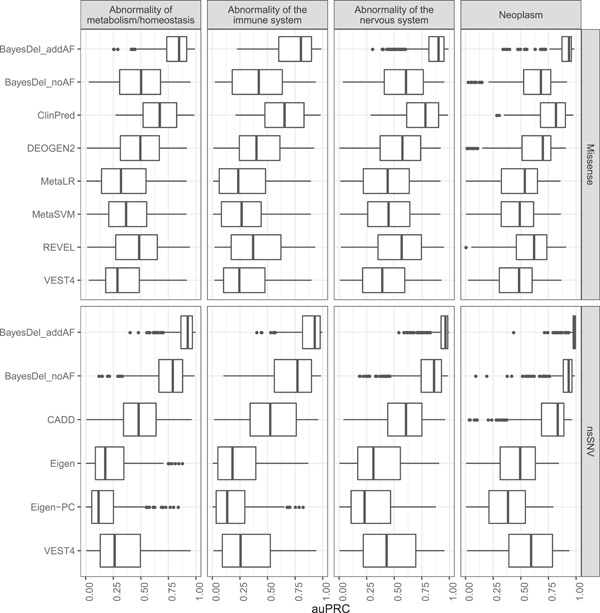
Boxplots showing auPRC of the top performing variant prioritization tools across selected top level HPO phenotypic abnormality terms and all their descendant terms for missense variants and nsSNVs. Abnormality of metabolism/homeostasis includes 340 terms for missense variants and 443 for nsSNVs. Abnormality of the immune system includes 242 terms for missense variants and 288 for nsSNVs. Abnormality of the nervous system includes 806 terms for missense variants and 898 for nsSNVs. Neoplasm includes 227 terms for missense variants and 291 for nsSNVs. auPRC, area under the precision‐recall curve; nsSNV, nonsynonymous single nucleotide variant

When comparing the four top level HPO terms, strongest performance was seen across all tools for HPO terms associated with Neoplasm for both missense variants (median auPRC range: 0.48–0.94) and nsSNVs (median auPRC range: 0.38–0.99). For missense variants, all tools showed weakest performance for terms associated with Abnormality of the immune system (median auPRC range: 0.23–0.80). For nsSNVs, weakest performance was seen for terms associated with Abnormality of metabolism/homeostasis (median auPRC range: 0.12–0.93) and Abnormality of the immune system (median auPRC range: 0.13–0.93). In summary we found BayesDel_addAF to be the best performing tool for both missense variants and nsSNVs. Additionally, all tools exhibited stronger performance when prioritizing missense variants and nsSNVs for HPO terms associated with Neoplasm versus terms associated with abnormalities of metabolism/homeostasis, the immune system and the nervous system.

In summary, we found that the best performing variant prioritization tools differ depending on whether they are being used to prioritize missense variants or nsSNVs. Prioritization of missense variants is a more challenging task when compared to nonsense variants as nonsense variants usually affect protein function due to truncation. Whilst missense variants can also cause loss of protein function, the occurrence of this is rarer (around 20%) than that seen for nonsense variants (Kryukov et al., 2007).

The top performing tool in terms of auPRC for both missense variants and nsSNVs was BayesDel_addAF, with strongest performance seen for prioritization of nsSNVs. We also recommend ClinPred, the second best performer for missense variants as it showed consistent performance across a range of disease phenotypes. Whilst CADD was an overall weak performer for prioritizing missense variants, its overall performance for prioritizing nsSNVs was much improved. Hence, we also recommend CADD as a tool for prioritization of nsSNVs.

When considering performance based on PP25, BayesDel_addAF was again a top performer, consistently ranking ClinVar pathogenic variants within the top 25 ranked variants for both missense variants and nsSNVs across most HPO terms. However, in contrast to auPRC, strong performance was seen for conservation scores for both missense variants (LRT, phastCons100way and SIFT) and nsSNVs (LRT, phastCons30way, and phastCons100way). Similarly to the auPRC, CADD was also a strong performer for nsSNVs but not for missense variants.

Performance of the variant prioritization tools differs, even amongst the top performers, across the four top level HPO terms. Strongest performance for both missense variants and nsSNVs was seen for disease phenotypes associated with Neoplasm (HP:0002664). This is likely due to cancer being a more common disease that is better studied than rare diseases associated with Abnormality of metabolism/homeostasis (HP:0001939), Abnormality of the immune system (HP:0002715) and Abnormality of the nervous system (HP:0000707). This means pathogenic variants related to cancer will be overrepresented when compared to rarer diseases and hence also be overrepresented in training datasets of machine learning and ensemble methods. Furthermore, this points to the importance of developing tools that prioritize variants in a disease aware manner rather than the agnostic approach of the tools assessed here (Masica & Karchin, 2016).

In line with estimates of auPRC from our previous benchmark study (Anderson & Lassmann, 2018), we find that machine learning scores and ensemble scores show far superior performance than conservation scores when prioritizing variants across disease phenotypes. However, we do note that the training datasets used by machine learning and ensemble methods overlap in terms of the variants being assessed in this benchmark. This will result in more optimistic auPRC values for these methods in comparison to conservation methods. BayesDel and ClinPred in particular were trained on ClinVar pathogenic variants and given that our benchmark includes the same variants this will be contributing to their strong performance. Therefore, we cannot comment on whether the performance generalizes to yet unseen variants. Regardless of this, machine learning and ensemble methods can be expected to be superior to conservation methods as the pathogenicity of a variant can be predicted based on data that does not directly relate to conservation. Our benchmark is pragmatic in the sense that we focus on how these tools perform when used “out of the box” for the task of prioritizing variants. Though we do not recommend conservation measures based on the auPRC, some did perform well based on the PP25. In particular, LRT and phastCons100way were both strong performers for missense variants and nsSNVs.

In summary, we recommend use of BayesDel_addAF for prioritization of missense variants and nsSNVs. Given that in silico prediction tools have not reached the level of robustness required for clinical diagnostics (Richards et al., 2015; Strande et al., 2018), we further recommend use of ClinPred and CADD alongside BayesDel_addAF when prioritizing missense variants and nsSNVs respectively. BayesDel_addAF is also recommended for those who wish to examine a small number of top ranked missense variants or nsSNVs and for this task we further recommend simultaneous ranking with either LRT or phastCons100way. Of the five top performers we previously recommended (FATHMM, M‐CAP, MetaLR, MetaSVM, and VEST3) (Anderson & Lassmann, 2018), MetaLR, MetaSVM and VEST4 (updated from VEST3) were amongst the top performing tools but their performance has been surpassed by new tools included in the current benchmark. The task of prioritizing variants remains a challenge, however the tools recommended here should prove useful for reducing the number of variants for follow up and ultimately contribute to disease diagnosis.

## 2 METHODS

We previously described in detail our automated pipeline to integrate phenotypes with annotated variants (Anderson & Lassmann, 2018). Therefore, we only briefly describe each component and focus on describing updates to the benchmark.

### 2.1 Human phenotype ontology

We used package ontologyIndex (Greene et al., 2017) within R 3.6.3 (R Core Team, 2020) to read in and process the HPO (Köhler et al., 2014) (HPO) obo file which was downloaded from http://purl.obolibrary.org/obo/hp.obo on the 28th of January 2021. We retrieved all 15,290 descendant terms of the Phenotypic abnormality (HP:0000118) term using the get_descendants() function.

### 2.2 Linking disease phenotypes to genes using phenolyzer

Phenolyzer (Yang et al., 2015) was used to generate gene lists for the 15,290 HPO terms obtained above (File S1). We used the command line version available at https://github.com/WGLab/phenolyzer with default settings (i.e., options ‐p ‐ph ‐logistic ‐addon DB_DISGENET_GENE_DISEASE_SCORE,DB_GAD_GENE_DISEASE_SCORE ‐addon_weight 0.25).

### 2.3 Linking candidate genes to causative variants using dbNSFP annotations

The database for nonsynonymous SNPs’ functional predictions (dbNSFP) contains annotation for 84,013,490 potential nsSNVs and splicing‐site SNVs in the human genome (Liu et al., 2011; Liu et al., 2020). We used dbNSFP version 4.1a (release 16 June, 2020) which is based on Gencode release 29/Ensembl version 94 (Cunningham et al., 2019; Frankish et al., 2019). We selected all variants occurring in the gene lists returned by Phenolyzer. We restricted our analysis to ClinVar (Landrum et al., 2016) “pathogenic” variants that were associated with a single gene. In total we obtained 35,167 pathogenic variants linked to genes associated with disease phenotypes (File S2). Of these, 16,411 were nonsense variants and 18,756 were missense variants.

### 2.4 Benign variants

We used a set of 63,197 common (allele frequency ≥1% and <25%) missense variants obtained from the Exome Aggregation Consortium (ExAC) database (Niroula & Vihinen, 2019). These variants were downloaded from VariBench (Sasidharan Nair & Vihinen, 2013) (http://structure.bmc.lu.se/VariBench/ExAC_AAS_20171214.xlsx) and annotated with dbNSFP. We removed variants with ClinVar annotation other than “benign” and variants associated with more than one gene. We further filtered the variants to those 29,173 that had scores across all variant prioritization tools and used these in the benchmark analysis (File S3).

### 2.5 Performance evaluation

For each HPO term, we evaluated the performance of variant prioritization tools by assessing their ability to separate ClinVar pathogenic variants from benign variants. These assessments were performed separately for nsSNVs and missense variants (i.e., excluding nonsense variants) as not all tools score nonsense variants. We required each HPO term to be associated with at least 25 pathogenic variants and to have complete scores across all tools. In total, for missense variants we tested 37 tools across 4890 HPO terms and for nsSNVs we tested 22 tools across 5723 HPO terms.

We assessed the following 22 variant prioritization tools that score nsSNVs: BayesDel (with and without allele frequency) (Feng, 2017), bStatistic (McVicker et al., 2009), CADD (Kircher et al., 2014; Rentzsch et al., 2019), DANN (Quang et al., 2015), Eigen (Ionita‐Laza et al., 2016), Eigen‐PC (Ionita‐Laza et al., 2016), fathmm‐MKL (Shihab et al., 2015), fathmm‐XF (Rogers et al., 2018), fitCons‐i6 (Gulko et al., 2015), GenoCanyon (Lu et al., 2015), GERP++ (Davydov et al., 2010), LRT (Chun & Fay, 2009), MutationTaster (Schwarz et al., 2014), phastCons (17way_primate, 30way_mammalian, 100way_vertebrate) (Siepel et al., 2005), phyloP (17way_primate, 30way_mammalian, 100way_vertebrate) (Siepel et al., 2006), SiPhy (Garber et al., 2009) and VEST4 (Carter et al., 2013). Additionally, we assessed a further 15 tools that only score missense variants: ClinPred (Alirezaie et al., 2018), DEOGEN2 (Raimondi et al., 2017), FATHMM (Shihab et al., 2013), LIST‐S2 (Malhis et al., 2020), MetaLR (Dong et al., 2015), MetaSVM (Dong et al., 2015), MPC (Samocha et al., 2017), MutationAssessor (Reva et al., 2011), Polyphen2 (HDIV and HVAR) (Adzhubei et al., 2010), PrimateAI (Sundaram et al., 2018), PROVEAN (Choi et al., 2012), REVEL (Ioannidis et al., 2016), SIFT (Sim et al., 2012) and SIFT4G (Vaser et al., 2016). Further detail on the aforementioned tools is available in Table S1 of the dbNSFP v4 publication (Liu et al., 2020). We used the dbNSFP converted rank scores for each tool. We did not assess LINSIGHT (Huang et al., 2017) as this tool is focussed on prioritization of noncoding variants. We also omitted M‐CAP (Jagadeesh et al., 2016), MutPred (Pejaver et al., 2020) and MVP (Qi et al., 2021) as these tools were missing scores for a substantial proportion of the benign variants.

We used R package PRROC (Keilwagen et al., 2014) to calculate the area under the precision recall curve (auPRC) based on the interpolation of Davis and Goadrich (Davis & Goadrich, 2006). We also constructed another performance measure called PP25 that calculates the proportion of ClinVar pathogenic variants in the top 25 ranked variants. Whilst the auPRC quantifies how well each tool can separate pathogenic variants from the whole set of benign variants, PP25 focuses on how well each tool does in ranking pathogenic variants amongst the top 25 most pathogenic. Heatmaps of performance (auPRC) were produced using the R NMF package (Gaujoux & Seoighe, 2010).

## CONFLICTS OF INTEREST

The authors declare no conflicts of interest.

## AUTHOR CONTRIBUTIONS

**Denise Anderson**: performed analysis, interpreted results and drafted the manuscript. **Timo Lassmann**: conceived the study, interpreted results and drafted the manuscript.

## Supporting information

Supporting information.

Supporting information.

Supporting information.

Supporting information.

Supporting information.

Supporting information.

Supporting information.

Supporting information.

## Data Availability

The data that support the findings of this study are available in Files S1, S2, and S3. Code used to generate results for this study is available as File S4.
